# *In Vitro* Cytotoxicity of Reproductive Stage *Withania somnifera* Leaf and Stem on HepG2 Cell Line

**DOI:** 10.1155/2023/8832166

**Published:** 2023-12-26

**Authors:** Lali Lingfa, Aravinda Tirumala, Srinivas Ankanagari

**Affiliations:** ^1^Department of Genetics and Biotechnology, University College of Science, Osmania University, Hyderabad, India; ^2^Department of Botany, Nagarjuna Government College, Mahatma Gandhi University, Hyderabad, India

## Abstract

**Background:**

The ayurvedic plant *Withania somnifera*, a member of the Solanaceae family, has been used as a remedy for diverse health problems, including cancer.

**Objectives:**

The objective of this investigation was to conduct a comparative analysis of the *in vitro* cytotoxic properties of methanolic extracts derived from the leaf, stem, and root of *W. somnifera* on HepG2 and L929 cell lines.

**Methods:**

Methanolic extracts were obtained using the Soxhlet extraction method. To assess the *in vitro* anticancer action on the HepG2 and L929 cell lines, an MTT assay was performed. Changes in cell morphology were observed using an inverted microscope.

**Results:**

The MTT assay results indicated that the leaf, stem, and root methanolic extracts of *W. somnifera* showed significantly higher *in vitro* cytotoxicity in HepG2 cells, with IC_50_ values of 43.06 ± 0.615, 45.60 ± 0.3, and 314.4 ± 0.795 *μ*g/mL than in L929 cell lines with 78.77 ± 0.795, 90.55 ± 0.800, and 361.70 ± 0.795 *μ*g/mL, respectively. The leaf methanolic extract was the most effective, followed by the stem methanolic extract in the HepG2 cell line.

**Conclusion:**

The results of our study have confirmed that the methanolic extracts of both the leaf and stem of *W. somnifera* exhibit significant *in vitro* cytotoxicity in HepG2 cell lines, while displaying no significant cytotoxicity in the L929 cell line. Furthermore, the data obtained from the MTT assay indicate that the leaf methanolic extract possesses a more potent cytotoxic activity than the stem methanolic extract with respect to the HepG2 cell line. Further studies on the identification and isolation of bioactive metabolites are required to explore the mechanisms underlying their *in vitro* cytotoxicity.

## 1. Introduction

Ayurvedic medicines have long been used with various medicinal plants and their parts in several medicinal formulations [1]. *Withania somnifera* is an important herb that belongs to the Solanaceae family. It is also known as Indian ginseng, winter cherry, or ashwagandha [2]. Indians have been using this herb for therapeutic purposes since time immemorial [3]. However, systemic scientific research on this plant began in the 1950s only [4]. Initially, it was used to treat fertility and reproductive health issues, but it is now used to prevent aging, boost important body fluids such as blood cells, lymph secretion, semen, and nourish various body organs [5].

Despite advancements in medicine, cancer remains the main cause of mortality worldwide [6]. Among noncommunicable diseases, cancer is the second leading cause of death [7]. Uncontrolled cell proliferation, metastasis, invasion, programmed cell death, and angiogenesis are hallmarks. Cancer can affect any organ in the human body, although it most frequently affects the breast, lung, liver, colon, prostate, kidney, and ovaries [8]. Approximately 29% of all malignancies are lung cancers [9]. Breast cancer is the major cause of morbidity in women, whereas prostate cancer is the major cause of morbidity in men. Liver cancer ranks third in terms of cancer mortality [10]. The rate of death from cancer is constantly increasing [11]. The lack of efficient anticancer drugs is a major burden on the healthcare system, and there is an urgent need for such drugs [12].

Despite the availability of many chemoprotective medications for cancer treatment, these are expensive and have numerous side effects [13]. Therefore, it is critically important to search for cost-effective and promising natural medications with minimal side effects to reduce cancer morbidity rates. Herbal medicines are considered the most viable ways to cure cancer [14]. A variety of plants, such as *W. somnifera,* are used for drug development. These are more effective and have fewer adverse effects. Thus, there is a pressing demand for natural medications to stop cancer progression and spread throughout the body [15].

*W. somnifera* has received a lot of attention recently for its anticancer studies [16]. *W. somnifera* extracts have been investigated from various plant parts for therapeutic value in treating cancers of various origins [17]. In one study, a decrease in mammary carcinomas in mice was observed with *W. somnifera* root extract [15]. In another study, human umbilical vein endothelial cells (HUVECs) were treated with *W. somnifera* root extract, and withaferin A reduced cell proliferation [18]. *W. somnifera* leaf extracts have been employed as a treatment during research investigations exploring its potential anticancer effects [15].

Previous studies have shown that *W. somnifera* extracts reduce the proliferation of MCF-7, pancreatic, prostate, kidney, and fibrosarcoma cells [19]. Consequently, it can be concluded that substances extracted from *W. somnifera* have strong antineoplastic activity and could potentially be used as chemotherapeutic agents. Moreover, several investigations have demonstrated the anticancer activities of *W. somnifera* during the course of research on the anticancer activities of this plant for the development of herbal-based drugs [20]. The 3-(4,5-dimethylthiazol-2-yl)-2,5-diphenyltetrazolium bromide (MTT) colorimetric method is a rapid and effective assay for assessing cellular metabolic activity, cytotoxicity, and proliferation [21]. In light of the comprehensive assessments documenting the anticancer attributes of *W. somnifera* [15], this article presents *in vitro* comparative investigations into the anticancer potentials of distinct constituents of *W. somnifera*, specifically the leaf, stem, and root, in the selected cell lines HepG2 and L929.

## 2. Materials and Methods

### 2.1. Chemicals

All chemicals and media were procured from Hi-Media and Sigma-Aldrich, respectively.

### 2.2. Cell Lines

The human liver cancer cell line HepG2 and the mouse fibroblast noncancerous cell line L929 were acquired from the National Centre for Cell Sciences (NCCS), Pune. The cell lines were later propagated in Dulbecco's Modified Eagle Medium (DMEM), supplemented with 10% (v/v) fetal bovine serum (FBS), an antimycotic antibiotic, and maintained with a continuous supply of 5% CO_2_ at 37°C.

### 2.3. Plant Material Collection

The *W. somnifera* seeds were bought from Zooqa Herbs, Chennai, Tamil Nadu. The seeds were sown and matured under natural soil and light conditions at the Department of Genetics and Biotechnology, Osmania University, Hyderabad. The plant materials were collected in the months of July–October 2022, during the monsoon season. The collected plant material was thoroughly washed three times with running water. Then, the plant material was rinsed once with sterile distilled water and weighted. The plant material was air dried in the shade at room temperature and weighed again. The fresh sample weight to dried sample weight ratios 37.02 : 23.82 of leaf, 28.72 : 16.92 of stem, and 28.31 : 14.71 of root, respectively, were used to coarsely grind and pulverise.

### 2.4. Plant Authentication

The authentication of *W. somnifera* was carried out by Dr. A. Vijaya Bhaskar Reddy, Botany Department, Osmania University, Hyderabad. The plant was deposited in the herbarium of the Botany Department, Osmania University, Hyderabad, with voucher number GEN/OU/001-2018-HY.

### 2.5. Plant Extraction

The leaf, stem, and root parts of *W. somnifera* were grounded coarsely and extracted with methanol in a Soxhlet for 24 hours and then air-dried. A viscous semisolid mass was produced using a rotary evaporator to concentrate the extract under reduced pressure at 40°C.

### 2.6. Culture Media

HepG2 and L929 cells were grown in Dulbecco's modified Eagle's medium (DMEM) (Himedia) containing streptomycin (100 *μ*g/mL) and penicillin (100 *μ*g/mL), supplemented with 10% (v/v) FBS. Cells were cultured at 37°C and 5% CO_2_; the complete medium was changed every three days (21, 22).

### 2.7. Morphological Study

In this study, cells in 6-well plates were used to examine the morphology of the cell lines *in vitro* under an inverted microscope.

### 2.8. *In Vitro* Evaluation of Cell Viability and Cytotoxicity

**Figure 1:**
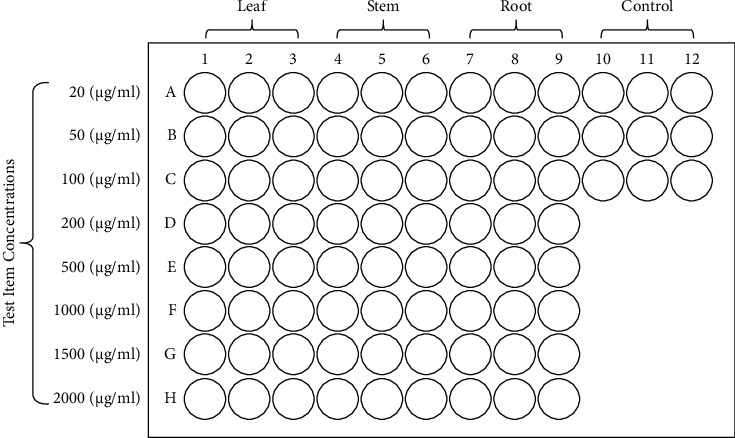
Microplate layout of HepG2 and L929 cell lines treated with 20, 50, 100, 200, 500, 1000, 1500, and 2000 *μ*g/mL of leaf, stem, and root methanolic extracts prepared from *W. somnifera*.

Cell viability and *in vitro* cytotoxicity were evaluated using an MTT assay [21–23]. In order to ascertain the impact of the methanolic leaf, stem, and root extracts of *W. somnifera* on HepG2 and L929 cells, an MTT assay was conducted. In 96-well plates, 100 *μ*L of each cell line was seeded at a density of 10,000 cells per well. The plates were then incubated for 48 h at 37°C in a 5% CO_2_ environment. After incubation, the cells were examined in a half-confluent monolayer. Next, 20, 50, 100, 200, 500, 1000, 1500, and 2000 *μ*g/mL of *W. somnifera* leaf, stem, and root methanolic extracts were treated in triplicate, as presented in Figure 1. The cells were then incubated at 37°C and 5% CO_2_ for 24 h.

The untreated cell lines served as the negative control, and the cells treated with the anticancer drug doxorubicin served as the positive control. Cell lines treated with the extracts served as test samples. After 24 hours, the cells were observed using an inverted microscope to check for any morphological changes or cell death. After observation, the culture medium was removed, and 100 µL of fresh medium was added along with 10 µL of MTT reagent (5 mg/mL). The plates were then placed in an incubator with 5% CO_2_ at 37°C for 4 h. Subsequently, the medium containing MTT was removed, the formazan purple precipitate was solubilized, and 100 µL of DMSO was added. The plates were then incubated for 1 hour at 37°C in a 5% CO_2_ incubator. The absorbance at 570 nm was measured using a Multiskan SkyHigh Plate Reader by subtracting the absorbance at 630 nm from the background after the purple formazan crystals completely dissolved. Using GraphPad Prism Version 8.0, a log graph of the log test item concentration *vs*. cell survival percentage was plotted and the half-maximal inhibitory concentration (IC_50_) values were calculated. [21, 23].

(1) $$\begin{array}{c} \text{Percent cell survival }\left(\%\right)=\frac{\text{Absorbance of Test}}{\text{Absorbance of Control}}X\;100. \end{array}$$

### 2.9. Statistical Analysis

All statistical analyses were carried out using the SPSS® statistical software package for Windows®, version 15.0 of SPSS Inc. (Chicago, IL, USA). The results are presented as means ± SD, and *p* ≤ 0.05 was used to determine whether treatment differences were significant.

## 3. Results

The MTT assay is widely used to assess cell viability, cell proliferation, cytotoxicity testing, and drug screening [21]. In drug screening, the MTT assay is used to test the cytotoxicity of various compounds, such as drugs, natural products, or experimental molecules, on cell lines or primary cells [21]. It measures the reduction of the MTT reagent to formazan, a purple-colored product indicating the metabolic activity of viable cells and the intensity of which is directly proportional to the number of viable cells in the sample [22]. Thus, it helps to identify compounds that inhibit cell growth or induce cell death. In this study, the comparative evaluation for anticancer potential of *W. somnifera* leaf, stem, and root methanolic extracts was carried out to determine their cytotoxic effect on the selected cancer cell line HepG2 and normal cell line L929 viability.

### 3.1. *In Vitro* Evaluation of Morphological Changes in HepG2 and L929 Cells

**Figure 2:**
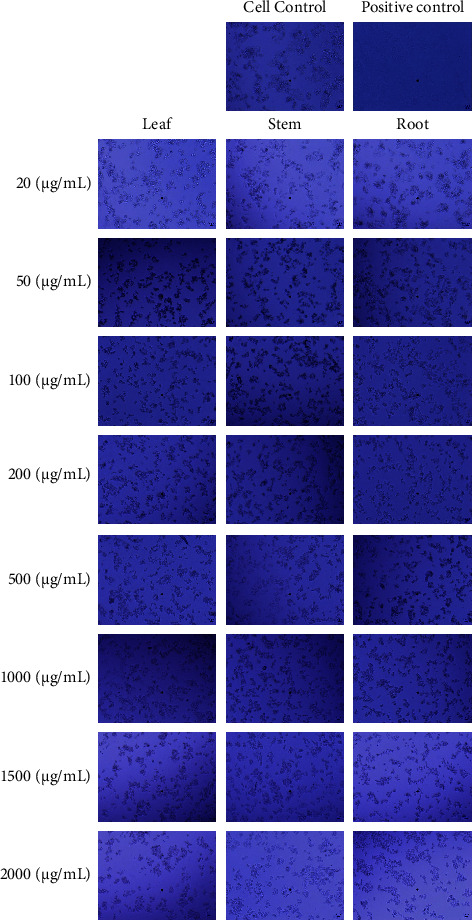
*In vitro* morphological profile of HepG2 cell observed under an inverted microscope at various concentrations compared to the controls (without the test sample and with the standard drug doxorubicin), after 24 hours of treatment with *W. somnifera* leaf, stem, and root methanolic extracts.

**Figure 3:**
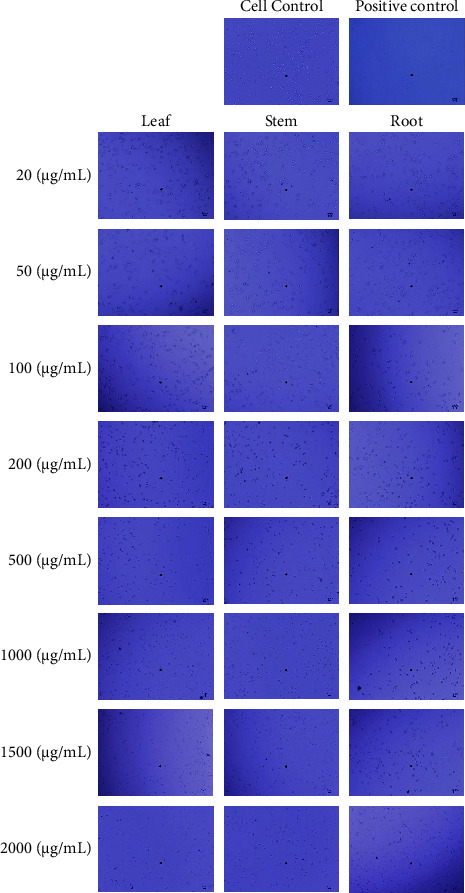
*In vitro* morphological profile of L929 cell observed under an inverted microscope at various concentrations compared to the controls (without the test sample and with the standard drug doxorubicin), after 24 hours of treatment with *W. somnifera* leaf, stem, and root methanolic extracts.

HepG2 and L929 cells were treated with *W. somnifera* leaf, stem, and root methanolic extracts of 20, 50, 100, 200, 500, 1000, 1500, and 2000 *μ*g/mL for 24 h. Figures 2 and 3 show the *in vitro* morphological profiles of HepG2 and L929 cells, respectively, under an inverted microscope. The treatments were compared to controls (a negative sample without a test sample and a positive sample with the standard drug doxorubicin). Cell morphology revealed significant morphological changes in HepG2 and L929 cells. The HepG2 cells showed adhesion to the well wall with *W. somnifera* methanolic extracts of root at 20 *μ*g/mL, leaf at 20 *μ*g/mL, and stem at 50 *μ*g/mL. On the other hand, L929 cells showed adherence to the well plate wall with *W. somnifera* methanolic extracts of root at 100 *μ*g/m L, leaf at 100 *μ*g/mL, and stem at 200 *μ*g/mL. However, at higher concentrations, the HepG2 and L929 cells appeared to be in a state of splitting, detaching from the substrate, and increasing the number of suspended cells.

### 3.2. *In Vitro* Evaluation of HepG2 and L929 Cell Lines Viability

The viability of HepG2 and L929 cells was evaluated using the MTT assay. A considerable reduction in cell viability was observed in HepG2 (Tables 1–3) in a concentration-dependent manner. At the highest 2000 *μ*g/mL concentration of *W. somnifera* leaf, stem, and root methanolic extracts, MTT assay results indicated that 100% viability in control HepG2 cells decreased to 10.19 ± 0.015%, 9.93 ± 0.051%, and 9.82%, respectively (Tables 1–3). Moreover, the viability of HepG2 cells at the lowest concentration of 20 *μ*g/mL *W. somnifera* methanolic leaf, stem, and root extracts was observed to be 81.94 ± 0.046%, 86.09 ± 0.020%, and 95.99 ± 0.025%, respectively. These values were much lower than the viability of the control cells (untreated cells plus media), which ranged between 99 and 100%.

Similarly, at the highest 2000 *μ*g/mL concentration of *W. somnifera* leaf, stem, and root methanolic extracts, the viability of L929 cells decreased from 100% in controls to 26.53 ± 0.045%, 25.94 ± 0.026%, and 26.87 ± 0.058%, respectively (Tables 4–6). In contrast, at the minimal concentration of 20 *μ*g/mL *W. somnifera* leaf, stem, and root methanolic extracts, the viability of L929 cells was 72.37 ± 0.030%, 71.97 ± 0.052%, and 77.96 ± 0.030%, respectively. These values were greater than the values of HepG2 cell viability. However, these values were much lower than the 99–100% viability of the control cells (untreated cells + media). Accordingly, the decrease in viability of HepG2 cells was significant at *p* ≤ 0.05, indicating the effective cytotoxicity of the *W. somnifera* leaf and stem methanolic extracts. Interestingly, the extracts did not considerably affect L929 cells.

### 3.3. IC_50_ Evaluation of Methanolic Leaf, Stem, and Root Extracts

**Figure 4:**
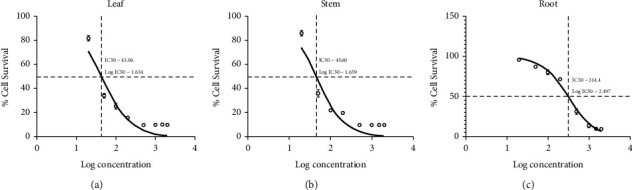
IC_50_ values in the HepG2 cell line treated with *W. somnifera* methanolic extracts from (a) leaf, (b) stem, and (c) root.

**Figure 5:**
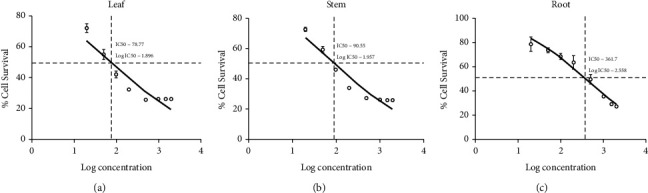
IC_50_ values in the L929 cell line treated with *W. somnifera* methanolic extracts (a) leaf, (b) stem, and (c) root.

The *in vitro* cytotoxicity of *W. somnifera* leaf, stem, and root methanolic extracts was assessed using the HepG2 and L929 cell lines, with the aim of determining their IC_50_ values (Figures 4 and 5). The IC_50_ values for the methanolic extracts of *W. somnifera* leaf and stem were determined to be 43.06 ± 0.615 *μ*g/mL and 45.60 ± 0.3 *μ*g/mL, respectively, in HepG2 cell lines. In the L929 cell lines, the IC_50_ values were found to be 78.77 ± 0.795 *μ*g/mL and 90.55 ± 0.800 *μ*g/mL for the leaf and stem extracts, respectively. On the other hand, IC_50_ values with root were 314.4 ± 0.795 and 314.4 ± 0.795 *μ*g/mL in the HepG2 and L929 cell lines, respectively. Overall, the IC_50_ values obtained for *W. somnifera* leaf and stem methanolic extracts in HepG2 were less than 50.00 *μ*g/mL and greater than 50.00 *μ*g/mL in the L929 cell line. The IC_50_ values obtained for the root were greater than 100 *μ*g/mL in both cell lines, HepG2 and L929. By calculating “*p*” values, the significance of difference between the observed value and the hypothesized mean of IC_50_ was determined for leaf, stem, and root methanolic extracts of W*. somnifera* in HepG2 and L929 cells (Table 7). The *p* values for IC_50_ of *W. somnifera* leaf, stem, and root methanolic extracts were found to be 0.0014, 0.0007, and 0.9999 in HepG2 and 0.9993, 0.9994, and 0.9993 in L292 cell lines, respectively.

## 4. Discussion

*W. somnifera* is a reliable source of herbal medicinal products [24]. Previous studies on *W. somnifera* leaf and root methanolic extracts have indicated that they are a source of novel phytochemicals that can inhibit cancer [25–28]. Furthermore, studies showed effective anticancer activity of methanolic extracts of *W. somnifera* leaf against MDA-MB-231 [29], IMR-32 [30], MCF-7 (breast) [31, 32], stem against HCT-15 (colon), and root against A-549, DU-145 [30], and B16F1 [29] cell lines. Moreover, *W. somnifera* anticancer potential with reference to methanolic extracts of reproductive stage stem, leaf, and root fractions revealed an array of phytochemicals with anticancer properties being present [32, 33].

Human hepatocellular carcinoma (HCC), the most prevalent cancer with no effective treatment, is a malignant tumor that develops from hepatocytes [34]. Globally, it is the fifth most common cause of cancer and the second most common cause of cancer-related deaths [35]. In a previous study, HepG2 cells (a cell line obtained from hepatocellular carcinoma) were known to be arrested in the S phase of the cell cycle [36]. The MTT assay is a useful method to compare the cytotoxic activity of cancer cell lines to that of a normal cell line, L929 (mouse fibroblast normal cell line) [37]. Nevertheless, there exists a gap in the comparative analysis of the anticancer effects of *W. somnifera* on HepG2 and L929 cell lines, focusing on plant organ-based research. This gap persists despite the extensive utilization of *W. somnifera* in various studies pertaining to anticancer properties. Hence, in this study, the anticancer activities of *W. somnifera* leaf, stem, and root methanolic extracts were evaluated using the MTT assay in HepG2 and L929 cells.

The inverted microscope images of HepG2 and L929 cells (Figures 2 and 3) revealed significant dose-dependent morphological changes. The results of the experiment indicate that after a 24-hour treatment with methanolic extracts derived from the stem, leaf, and root of *W. somnifera*, HepG2 cells exhibited minimal morphological alterations at lower concentrations ranging from 20 to 50 *μ*g/mL, while L929 cells displayed negligible changes at higher concentrations ranging from 100 to 200 *μ*g/mL. This observation is in agreement with earlier findings that *W. somnifera* extracts exhibited less cytotoxicity against normal cell lines, such as L929, than against cancerous cell lines [38]. This may be attributed to the presence of certain phytochemicals in the extracts that selectively target cancer cells while sparing normal cells.

In both HepG2 and L929 cell lines at higher concentrations of *W. somnifera* stem, leaf, and root methanolic extracts, significant cell changes were observed, such as an increase in suspended cells, a decrease in cell density, reduced cell volume, detachment from the substrate, and cytoplasmic shrinkage. This result confirms earlier findings on HepG2 cells exhibiting characteristics of apoptosis *via* morphological changes when treated with *W. somnifera* extracts at different concentrations for 24 hours [39]. This suggests that *W. somnifera* stem and leaf methanolic extracts have anticancer properties, which may be due to bioactive compounds such as flavonoids, withanolides, and alkaloids that induce apoptosis [26]. The proportional morphological alterations observed in HepG2 cells upon exposure to increasing concentrations of methanolic extracts derived from the leaf, stem, and root of *W. somnifera* were found to be consistent with previous research, which demonstrated a dose-dependent inhibition of cell proliferation and associated morphological changes [38, 40]. The increased suppression of cellular proliferation observed at higher concentrations can be attributed to the existence of phytochemicals, which possess the capability to affect the structure and functionality of cancer cells. This impact may lead to morphological changes in HepG2 cancer cells, ultimately resulting in their death or hindering their ability to multiply and spread.

When treated with methanolic extracts of *W. somnifera* leaf and stem, the evaluation of viable cells using the MTT assay revealed a significant reduction in HepG2 cell viability in a dose-dependent manner. The HepG2 cell line exhibited cell viabilities of 81.94 ± 0.046, 34.25 ± 0.015, 25.62 ± 0.011, 16.07 ± 0.017, 10.05 ± 0.02, 10.19 ± 0.015, 10.25 ± 0.011, and 10.19 ± 0.015 at concentrations of 20, 50, 100, 200, 500, 1000, 1500, and 2000 *μ*g/mL with methanolic leaf extracts, as shown in Table 1. Similarly, HepG2 exhibited cell viability of 86.09 ± 0.020, 36.21 ± 0.011, 22.16 ± 0.017, 19.85 ± 0.015, 10.01 ± 0.025, 10.04 ± 0.025, and 10.04 ± 0 0.025, at 20, 50, 100, 200, 500, 1000, 1500, and 2000 *μ*g/mL concentrations, respectively, with stem extracts (Table 2). On the other hand, HepG2 exhibited cell viability of 9.93 ± 0.05 and 95.99 ± 0.025, 87.44 ± 0.015, 80.52 ± 0.020, 72.06 ± 0.017, 31.69 ± 0.017, 14.27 ± 0.025, 10.28 ± 0.035, and 9.82 ± 0.125 at 20, 50, 100, 200, 500, 1000, 1500, and 2000 *μ*g/mL concentrations with root methanolic extracts (Table 3). It has been determined that the methanolic extract derived from the leaf exhibited the highest level of activity followed by stem in inhibiting the viability of HepG2 cells, surpassing the methanolic extracts obtained from the root. This result supports previous findings that HepG2 cell viability decreases significantly as the *W. somnifera* leaf methanolic extract concentration increases [36]. The aforementioned data suggest a heightened efficacy of the methanolic extract derived from *W. somnifera* leaf in inhibiting the proliferation of the Hep G2 cell line.

In contrast to that of HepG2 cells, the viability of L929 cells was insignificant when compared in a dose-dependent manner. The L929 cell line exhibited cell viability of 99.99 ± 0.005, 72.37 ± 0.030, 55.39 ± 0.025, 42.38 ± 0.30, 32.6 ± 0.020, 26.25 ± 0.035, 26.53 ± 0.02, 26.59 ± 0.020, and 26.53 ± 0.045 at concentrations of 20, 50, 100, 200, 500, 1000, 1500, and 2000 *μ*g/mL with leaf methanolic extracts (Table 4). Similarly, L929 exhibits cell viabilities of 99.99 ± 0.005, 71.97 ± 0.052, 58.87 ± 0.040, 45.57 ± 0.037, 33.81 ± 0.045, 27.1 ± 0.068, 25.85 ± 0.037, 25.77 ± 0.055, and 25.94 ± 0.026; and 99.99 ± 0.005, 77.96 ± 0.030, 72.91 ± 0.0467.77 ± 0.060, 62.88 ± 0.045, 49.16 ± 0.030, 35.1 ± 0.041, 28.52 ± 0.040, and 26.87 ± 0.058 (Tables 5 and 6) at the 20, 50, 100, 200, 500, 1000, 1500, and 2000 *μ*g/mL concentrations with stem and root methanolic extracts, respectively. This finding indicates that the leaf methanolic extracts of *W. somnifera* exhibit anticancer properties compared to the stem and root methanolic extracts of the HepG2 cell line. This may be ascribed to certain bioactive compounds such as the phenolic compounds 1,2-bis (trimethylsilyl) benzene, the ester compound boric acid, trimethyl ester, and steroid amines such as dextroamphetamine [41] in the methanolic extract of *W. somnifera* leaf, which have been shown to inhibit the growth of cancer cells in the previous finding.

The findings from the cytotoxicity evaluations conducted using the MTT assay revealed that the IC_50_ values of methanolic extracts obtained from the leaf, stem, and root of *W. somnifera* exhibited significant potency in HepG2 cells. The IC_50_ values of *W. somnifera* leaf, stem, and root methanolic extracts in HepG2 cells were found to be 43.06 *μ*g/mL, 45.60 *μ*g/mL, and 314.4 *μ*g/mL (Figure 4), and L929 78.77 *μ*g/mL, 90.55 *μ*g/mL, and 361.70 *μ*g/mL (Figure 5), respectively. The IC_50_ value in pharmacological research is generally considered an indicator of a drug's effectiveness at its half-inhibitory concentration. It provides antagonist drug potency by demonstrating the amount of drug required to block 50% of the biological process [29]. Moreover, as per the GERAN Protocol and the American National Cancer Institute (NCI), IC_50_ values for strong cytotoxic properties are defined as under 21 *μ*g/mL, moderate cytotoxic properties between 21and 200 *μ*g/mL, and weak cytotoxic properties between 201 and 500 *μ*g/mL [42]. IC_50_ values greater than 50 *μ*g/mL are considered noncytotoxic [42, 43]. In this study, the IC_50_ values were found to be less than 50 *μ*g/mL for the leaf and stem methanolic extracts of HepG2 cells, which were found to be much lower than the IC_50_ values for L929 cells. An IC_50_ value of less than 50 *μ*g/mL is usually considered to have significant cytotoxicity [43]. The MTT assay findings of the methanolic leaf and stem extracts of *W. somnifera* indicate a significant degree of cytotoxicity on HepG2 cells, ranging from high to moderate, while demonstrating only weak cytotoxicity on the L929 cell line [9, 29, 43]. These results support earlier findings that *W. somnifera* leaf methanolic extracts are cytotoxic to HepG2 cells [44]. The methanolic extracts derived from the root of *W. somnifera* were found to lack satisfactory cytotoxic activity against both HepG2 and L929, as evidenced by IC_50_ values exceeding 100 *μ*g/mL. This observation is consistent with prior research indicating that *W. somnifera* root extract has a minimal cytotoxic impact on L929 cells [7, 9, 45]. This selective cytotoxic effect on cancer cells may be due to bioactive compounds in the methanolic extracts of *W. somnifera* that specifically target cancer cell lines, such as HepG2.

Statistical analysis showed a significant effect of *W. somnifera* leaf and stem methanolic extracts on HepG2 cells (*p* ≤ 0.05). The methanolic root extracts of *W. somnifera* did not exhibit any significant impact on either HepG2 or L929 cell lines, as indicated by the *p* value of ≥0.05 (Table 7). Similarly, leaf and stem methanolic extracts showed no significant effect on the L929 cell line (*p* ≥ 0.05). Thus, the *p* values further confirmed that the methanolic extracts of *W. somnifera* leaf and stem had significant cytotoxicity in HepG2 cells and weak cytotoxicity in the L929 cell line.

## 5. Conclusion

*W. somnifera* is a reliable source of herbal products. Research has revealed an array of anticancer phytochemicals present in the stem, leaf, and root. This study was conducted using the MTT assay to compare the cytotoxic effects of methanolic leaf, stem, and root extracts of *W. somnifera* on HepG2 and L929 cells. The results of this investigation demonstrated that the methanolic leaf and stem extracts of *W. somnifera* exhibited significant cytotoxicity in HepG2 cells, while no significant cytotoxicity was observed in L929 cells. Furthermore, the IC_50_ values indicated that the leaf methanolic extracts possessed the highest cytotoxic activity, followed by the stem methanolic extract in the HepG2 cell line. Hence, further studies on the identification and isolation of bioactive metabolites are required to explore the mechanism of cytotoxicity.

## Figures and Tables

**Table 1:** IC_50_ value in the HepG2 cell line treated with different concentrations of *W. somnifera* leaf methanolic extract.

Leaf methanolic extracts of *W. somnifera* on HepG2 cell lines			
Concentration (*μ*g/mL)	Absorbance	% cell survival	% inhibition
Control	1.143 ± 0.0005	100	0
20	0.9365 ± 0022	81.94 ± 0.046	18.06 ± 0.025
50	0.3915 ± 0.0004	34.25 ± 0.015	65.75 ± 0.028
100	0.2928 ± 0.0001	25.62 ± 0.011	74.38 ± 0.025
200	0.1837 ± 0.0002	16.07 ± 0.017	83.93 ± 0.015
500	0.1149 ± 0.0001	10.05 ± 0.02	89.95 ± 0.068
1000	0.1165 ± 0.0001	10.19 ± 0.015	89.81 ± 0.090
1500	0.1171 ± 0.0001	10.25 ± 0.011	89.75 ± 0.165
2000	0.1164 ± 0.0001	10.19 ± 0.015	89.81 ± 0.035
Log IC_50_ value	1.634 ± 0.0005		
IC_50_ value	**43.06** **±** **0.615 μg/mL**		

IC_50_ value in bold indicates significant at *p* ≤ 0.05.

**Table 2:** IC_50_ value in the HepG2 cell line treated with different concentrations of *W. somnifera* stem methanolic extract.

Effect of stem methanolic extract of *W. somnifera* in HepG2 cell line			
Concentration (*μ*g/mL)	Absorbance	% cell survival	% inhibition
Control	1.143 ± 0.0015	100	0
20	0.984 ± 0.0017	86.09 ± 0.020	13.91 ± 0.030
50	0.4139 ± 0.0001	36.21 ± 0.011	63.79 ± 0.025
100	0.2533 ± 0.0001	22.16 ± 0.017	77.84 ± 0.015
200	0.2269 ± 0.0001	19.85 ± 0.015	80.15 ± 0.020
500	0.1144 ± 0.0005	10.01 ± 0.025	89.99 ± 0.046
1000	0.1148 ± 0.0002	10.04 ± 0.025	89.96 ± 0.020
1500	0.1147 ± 0.0004	10.04 ± 0 0.025	89.96 ± 0.026
2000	0.1135 ± 0.0001	9.93 ± 0.051	90.07 ± 0.045
Log IC_50_ value	1.659 ± 0.0015		
IC_50_ value	**45.60** **±** **0.3 μg/mL**		

IC_50_ value in bold indicates significance at *p* ≤ 0.05.

**Table 3:** IC_50_ value in the HepG2 cell line treated with different concentrations of *W. somnifera* root methanolic extract.

Effect of root methanolic extract of *W. somnifera* in HepG2 cell line			
Concentration (*μ*g/mL)	Absorbance	% cell survival	% inhibition
Control	1.143 ± 0.0005	100	0
20	1.0972 ± 0.0003	95.99 ± 0.025	4.01 ± 0.068
50	0.9995 ± 0.0009	87.44 ± 0.015	12.56 ± 0.050
100	0.9203 ± 0.0114	80.52 ± 0.020	19.48 ± 0.040
200	0.8236 ± 0.0002	72.06 ± 0.017	27.94 ± 0.023
500	0.3622 ± 0.0004	31.69 ± 0.017	68.31 ± 0.015
1000	0.1631 ± 0.0006	14.27 ± 0.025	85.73 ± 0.011
1500	0.1175 ± 0.0002	10.28 ± 0.035	89.72 ± 0.015
2000	0.1122 ± 0.0002	9.82 ± 0.125	90.18 ± 0.025
Log IC_50_ value	2.497 ± 0.0015		
IC_50_ value	**314.4** **±** **0.795 μg/mL**		

IC_50_ value in bold indicates not significant at *p* ≤ 0.05.

**Table 4:** IC_50_ value in the L929 cell line treated with different concentrations of *W. somnifera* leaf methanolic extract.

Effect of leaf methanolic extract of *W. somnifera* in L929 cell line			
Concentration (*μ*g/mL)	Absorbance	% cell survival	% inhibition
Control	0.4387 ± 0.0002	99.99 ± 0.005	0
20	0.3175 ± 0.0003	72.37 ± 0.030	27.63 ± 0.015
50	0.243 ± 0.0056	55.39 ± 0.025	44.61 ± 0.020
100	0.1859 ± 0.0005	42.38 ± 0.30	57.62 ± 0.045
200	0.143 ± 0.0030	32.6 ± 0.020	67.40 ± 0.028
500	0.1152 ± 0.0001	26.25 ± 0.035	73.75 ± 0.035
1000	0.1164 ± 0.0003	26.53 ± 0.02	73.47 ± 0.032
1500	0.1166 ± 0.0001	26.59 ± 0.020	73.41 ± 0.030
2000	0.1164 ± 0.0003	26.53 ± 0.045	73.47 ± 0.045
Log IC_50_ value	1.896 ± 0.0050		
IC_50_ value	**78.77** **±** **0.795** **μg/mL**		

IC_50_ value in bold indicates not significant at *p* ≤ 0.05.

**Table 5:** IC_50_ value in the L929 cell line treated with different concentrations of *W. somnifera* stem methanolic extract.

Effect of stem methanolic extract of *W. somnifera* in L929 cell line			
Concentration (*μ*g/mL)	Absorbance	% cell survival	% inhibition
Control	0.4387 ± 0.0005	99.99 ± 0.005	0
20	0.3157 ± 0.0003	71.97 ± 0.052	28.03 ± 0.580
50	0.2583 ± 0.0004	58.87 ± 0.040	41.13 ± 0.030
100	0.1999 ± 0.0014	45.57 ± 0.037	54.43 ± 0.036
200	0.1483 ± 0.0007	33.81 ± 0.045	66.19 ± 0.055
500	0.1189 ± 0.0001	27.1 ± 0.068	72.9 ± 0.340
1000	0.1134 ± 0.0003	25.85 ± 0.037	74.15 ± 0.112
1500	0.1131 ± 0.0005	25.77 ± 0.055	74.23 ± 0.060
2000	0.1138 ± 0.0004	25.94 ± 0.026	74.06 ± 0.119
Log IC_50_ value	1.957 ± 0.004		
IC_50_ value	**90.55** **±** **0.800** **μg/mL**		

IC_50_ value in bold indicates not significant at *p* ≤ 0.05

**Table 6:** IC_50_ value in the L929 cell line treated with different concentrations of *W. somnifera* root methanolic extract.

Effect of root methanolic extract of *W. somnifera* in L929 cell line			
Concentration (*μ*g/mL)	Absorbance	% cell survival	% inhibition
Control	0.4387 ± 0.0004	99.99 ± 0.005	0
20	0.342 ± 0.0032	77.96 ± 0.030	22.04 ± 0.141
50	0.3199 ± 0.0011	72.91 ± 0.040	27.09 ± 0.045
100	0.2973 ± 0.0014	67.77 ± 0.060	32.23 ± 0.035
200	0.2759 ± 0.0022	62.88 ± 0.045	37.12 ± 0.020
500	0.2157 ± 0.0004	49.16 ± 0.030	50.84 ± 0.036
1000	0.1540 ± 0.0003	35.1 ± 0.041	64.9 ± 0.042
1500	0.1251 ± 0.0003	28.52 ± 0.040	71.48 ± 0.057
2000	0.1179 ± 0.0005	26.87 ± 0.058	73.13 ± 0.030
Log IC_50_ value	2.558 ± 0.003		
IC_50_ value	**361.70** **±** **0.795** **μg/mL**		

IC_50_ value in bold indicates not significant at *p* ≤ 0.05.

**Table 7:** Statistical analysis of IC_50_*p* values obtained with *W. somnifera* leaf, stem, and root methanolic extract treatments in HepG2 and L929 cell lines.

IC_50_ value	*Leaf*		*Stem*		*Root*	
	HepG2	L929	HepG2	L929	HepG2	L929
Mean ($\bar{x}$)	43.48	78.77	45.6	90.55	314.4	361.21
Standard deviation	0.615	0.795	0.3	0.8	0.305	0.66
Standard error mean	0.355	0.459	0.173	0.461	0.176	0.381
Hypothesized mean	50	50	50	50	50	50
*p* value	0.0014	0.9993	0.0007	0.9993	0.9999	0.9994

## Data Availability

The data that support the findings of this study are available from the corresponding author upon reasonable request.

## References

[1] Kumar S., Dobos G. J., Rampp T. (2017). The significance of Ayurvedic medicinal plants. *Evidence-Based Complementary and Alternative Medicine*.

[2] Singh N., Bhalla M., de Jager P., Gilca M. (2011). An overview on ashwagandha: a rasayana (rejuvenator) of ayurveda. *African Journal of Traditional, Complementary and Alternative Medicines: AJTCAM*.

[3] Joshi V. K., Joshi A. (2021). Rational use of ashwagandha in ayurveda (traditional Indian medicine) for health and healing. *Journal of Ethnopharmacology*.

[4] Aminov R. I. (2010). A brief history of the antibiotic era: lessons learned and challenges for the future. *Frontiers in Microbiology*.

[5] Roozbeh N., Amirian A., Abdi F., Haghdoost S. (2021). A systematic review on use of medicinal plants for male infertility treatment. *Journal of Family and Reproductive Health*.

[6] Sung H., Ferlay J., Siegel R. L. (2021). Global cancer statistics 2020: GLOBOCAN estimates of incidence and mortality worldwide for 36 cancers in 185 countries. *CA: A Cancer Journal for Clinicians*.

[7] Wen Y. F., Chen M. X., Yin G. (2021). The global, regional, and national burden of cancer among adolescents and young adults in 204 countries and territories, 1990-2019: a population-based study. *Journal of Hematology & Oncology*.

[8] Gutschner T., Diederichs S. (2012). The hallmarks of cancer: a long non-coding RNA point of view. *RNA Biology*.

[9] Kanwal M., Ding X. J., Cao Y. (2017). Familial risk for lung cancer. *Oncology Letters*.

[10] Mattiuzzi C., Lippi G. (2019). Current cancer epidemiology. *Journal of Epidemiology and Global Health*.

[11] Siegel R. L., Miller K. D., Fuchs H. E., Jemal A. (2021). Cancer statistics, 2021. *CA: A Cancer Journal for Clinicians*.

[12] Barrios C., de Lima Lopes G., Yusof M. M., Rubagumya F., Rutkowski P., Sengar M. (2023). Barriers in access to oncology drugs a global crisis. *Nature Reviews Clinical Oncology*.

[13] Mokhtari R. B., Homayouni T. S., Baluch N. (2017). Combination therapy in combating cancer. *Oncotarget*.

[14] Yin S. Y., Wei W. C., Jian F. Y., Yang N. S. (2013). Therapeutic applications of herbal medicines for cancer patients. *Evidence-based Complementary and Alternative Medicine*.

[15] Palliyaguru D. L., Singh S. V., Kensler T. W. (2016). Withania somnifera: from prevention to treatment of cancer. *Molecular Nutrition & Food Research*.

[16] Kashyap V. K., Peasah-Darkwah G., Dhasmana A., Jaggi M., Yallapu M. M., Chauhan S. C. (2022). Withania somnifera: progress towards a pharmaceutical agent for immunomodulation and cancer therapeutics. *Pharmaceutics*.

[17] Siegel R. L., Miller K. D., Wagle N. S., Jemal A. (2023). Cancer statistics. *CA: A Cancer Journal for Clinicians*.

[18] Mohan R., Hammers H. J., Bargagna-Mohan P. (2004). Withaferin A is a potent inhibitor of angiogenesis. *Angiogenesis*.

[19] Sultana T., Okla M. K., Ahmed M. (2021). Withaferin A: from ancient remedy to potential drug candidate. *Molecules*.

[20] Rai M., Jogee P. S., Agarkar G., Santos C. A. D. (2016). Anticancer activities of Withania somnifera: current research, formulations, and future perspectives. *Pharmaceutical Biology*.

[21] Riss T. L., Moravec R. A., Niles A. L. (2004). Cell viability assays. *Assay Guidance Manual*.

[22] Graidist P., Martla M., Sukpondma Y. (2015). Cytotoxic activity of Piper cubeba extract in breast cancer cell lines. *Nutrients*.

[23] van Meerloo J., Kaspers G. J., Cloos J. (2011). Cell sensitivity assays: the MTT assay. *Methods in Molecular Biology*.

[24] Afewerky H. K., Ayodeji A. E., Tiamiyu B. B. (2021). Critical review of the Withania somnifera (L.) Dunal: ethnobotany, pharmacological efficacy, and commercialization significance in Africa. *Bulletin of the National Research Centre*.

[25] Dredge K., Dalgleish A. G., Marriott J. B. (2003). Angiogenesis inhibitors in cancer therapy. *Current Opinion in Investigational Drugs*.

[26] Saleem S., Muhammad G., Hussain M. A., Altaf M., Bukhari S. N. A. (2020). Withania somnifera L.: insights into the phytochemical profile, therapeutic potential, clinical trials, and future prospective. *Iranian Journal of Basic Medical Sciences*.

[27] Yousefian Z., Hosseini B., Rezadoost H., Palazón J., Mirjalili M. H. (2018). Production of anticancerous compound Withaferin A from genetically transformed hairy root culture of Withania somnifera. *Natural Product Communications*.

[28] Tewari D., Chander V., Dhyani A. (2022). Withania somnifera (L.) Dunal: phytochemistry, structure-activity relationship, and anticancer potential. *Phytomedicine*.

[29] Srivastava A. N., Ahmad R., Khan M. A. (2016). Evaluation and comparison of the in vitro cytotoxic activity of Withania somnifera methanolic and ethanolic extracts against MDA-MB-231 and Vero Cell Lines. *Scientia Pharmaceutica*.

[30] Yadav B., Bajaj A., Saxena M., Saxena A. K. (2010). In Vitro anticancer activity of the root, stem and leaves of Withania Somnifera against various human cancer cell lines. *Indian Journal of Pharmaceutical Sciences*.

[31] Alfaifi M. Y., Saleh K. A., El-Boushnak M. A., Elbehairi S. E., Alshehri M. A., Shati A. A. (2016). Antiproliferative activity of the methanolic extract of withania somnifera leaves from faifa mountains, southwest Saudi arabia, against several human cancer cell lines. *Asian Pacific Journal of Cancer Prevention*.

[32] Jayaprakasam B., Zhang Y., Seeram N. P., Nair M. G. (2003). Growth inhibition of human tumor cell lines by withanolides from Withania somnifera leaves. *Life Sciences*.

[33] Singh N., Yadav S. S., Rao A. S. (2021). Review on anticancerous therapeutic potential of Withania somnifera (L.) Dunal. *Journal of Ethnopharmacology*.

[34] Vakili Zahir N., Nakhjavani M., Hajian P., Shirazi F. H., Mirzaei H. (2018). Evaluation of silibinin effects on the viability of HepG2 (human hepatocellular liver carcinoma) and HUVEC (human umbilical vein endothelial) cell lines. *Iranian Journal of Pharmaceutical Research*.

[35] Tamboli A. M., Wadkar K. A. (2022). Comparative cytotoxic activity of Convolvulus pluricaulis against human hepatoma cell line (HepG2) and normal cell line (L929) via apoptosis pathways by flow cytometry analysis. *Bulletin of the National Research Centre*.

[36] Venkatachalapathy D., Shivamallu C., Prasad S. K. (2021). Assessment of chemopreventive potential of the plant extracts against liver cancer using HepG2 cell line. *Molecules*.

[37] Samarghandian S., Boskabady M. H., Davoodi S. (2010). Use of in vitro assays to assess the potential antiproliferative and cytotoxic effects of saffron (Crocus sativus L.) in human lung cancer cell line. *Pharmacognosy Magazine*.

[38] Parhar M., Bhullar K. K. (2022). Comparative evaluation of cytotoxicity of withania somnifera extract, sodium hypochlorite and chlorhexidine on L929 cell lines- an in-vitro study. *Baba Farid University Dental Journal*.

[39] Abutaha N. (2015). In vitro antiproliferative activity of partially purified Withania somnifera fruit extract on different cancer cell lines. *J BUON*.

[40] Kaplan A. (2022). The nanocomposites designs of phytomolecules from medicinal and aromatic plants: promising anticancer-antiviral applications. *Beni-Suef University Journal of Basic and Applied Sciences*.

[41] Lingfa L., Ankanagari S. (2023). GC-MS Profiling of reproductive stage Withania somnifera for antimicrobial and anticancer phytochemicals. *Biomedical and Pharmacology Journal*.

[42] Thanuja B., Parimalavalli R., Vijayanand S. (2022). Anticancer and cytotoxicity activity of native and modified black rice flour on colon cancer cell lines. *Evidence-based Complementary and Alternative Medicine*.

[43] Wang Y. T., Yang C. H., Huang T. Y., Tai M. H., Sie R. H., Shaw J. F. (2019). Cytotoxic effects of chlorophyllides in ethanol crude extracts from plant leaves. *Evidence-Based Complementary and Alternative Medicine*.

[44] Desai A. G., Qazi G. N., Ganju R. K. (2008). Medicinal plants and cancer chemoprevention. *Current Drug Metabolism*.

[45] Sudeep H. V., Gouthamchandra K., Venkatesh B. J., Prasad K. S. (2018). Viwithan, A standardized Withania somnifera root extract induces apoptosis in murine melanoma cells. *Pharmacognosy Magazine*.

